# When AI Starts Reading Battery Papers Like a Researcher

**DOI:** 10.1021/acscentsci.6c00760

**Published:** 2026-05-13

**Authors:** Dian-Zhao Lin, Yayuan Liu

**Affiliations:** Department of Chemical and Biomolecular Engineering, 1466 Johns Hopkins University, Baltimore, Maryland 21218, United States

## Abstract

A new AI agent mines both text and figures from lithium-metal-battery papers, pointing toward a future where the literature itself helps guide discovery.

## Graphical abstract

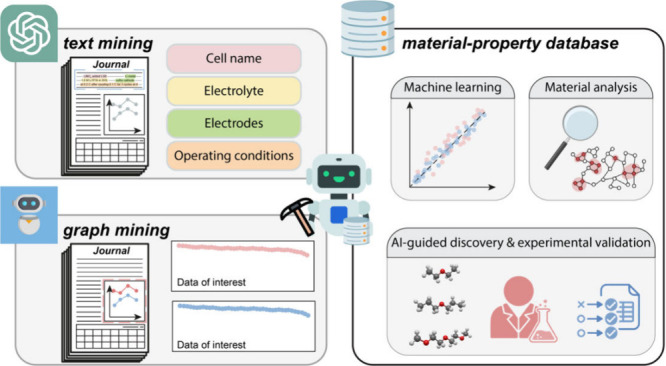

Researchers have long turned to the literature to inspire new experiments. However, as the volume of published work grows, actionable knowledge is increasingly fragmented across thousands of papers. Lithium metal batteries exemplify this challenge. Despite their promise for high-energy storage,^1^ progress in the field is slowed by a nearly combinatorial explosion of choices: parameters such as cathodes, electrolytes, salts, solvents, separators, current densities, cutoff voltages, and cycling protocols all interact to shape performance. Even for experts, it can be difficult to distinguish robust design rules from trends hiding inside a fragmented literature. In a recent issue of *ACS Central Science*,^2^ Kim and co-workers develop an AI agent to help researchers navigate an increasingly overwhelming literature.

Artificial intelligence (AI) has emerged as a powerful tool for materials discovery by mining patterns from scientific literature.^3^ However, previous efforts in battery research have largely focused on extracting characteristics of individual components from text, often overlooking performance data embedded in figures.^4^ The AI agent developed by Kim and co-workers, called LLMB (Large Language Model for Battery), addresses this gap by coupling hierarchical text mining with a graph-mining tool called Material Graph Digitizer (MatGD; Figure 1). This multimodal architecture is important because battery knowledge is inherently dispersed in scientific publications: key details are often buried in methods sections, while the most informative performance metrics may appear only in cycling plots. By treating both text and figures as data sources, the LLMB workflow comes closer to how scientists extract knowledge from papers in practice. Using this pipeline, the authors extracted compositional and operating condition data for 15,398 battery cells, digitized cyclability data for 10,242 cells, and ultimately constructed a curated database of 8,074 cells, achieving an accuracy of 0.989 and an F1 score of 0.933. By aligning textual and graphical information, the system begins to bridge a longstanding divide between what is described and what is measured in the literature.

**Figure 1:**
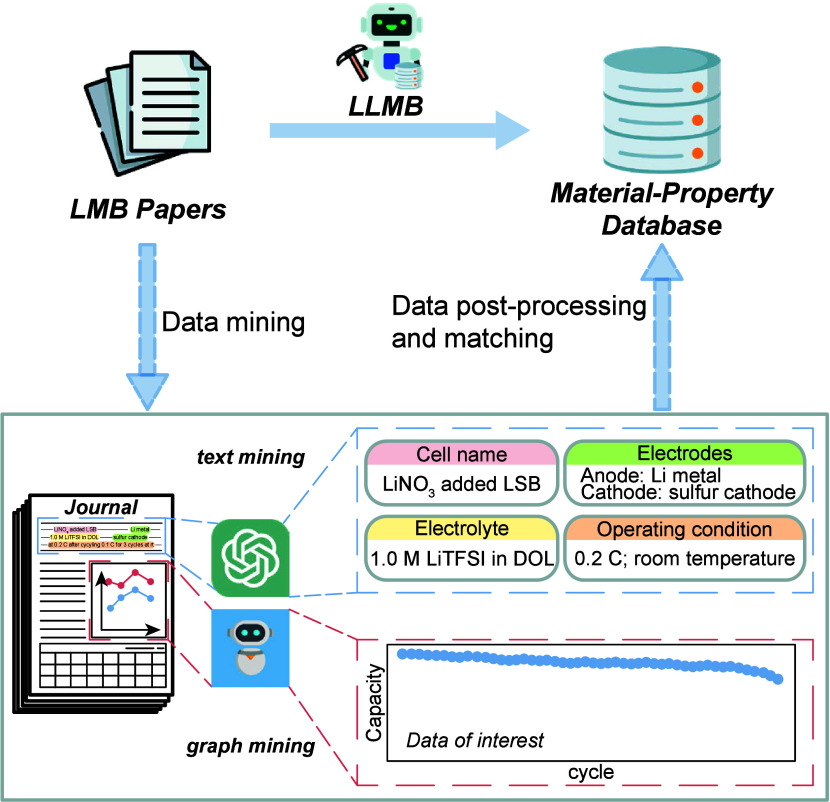
Conceptual workflow of LLMB. LLMB integrates a text-mining engine and a graph-mining tool to extract information from both text and battery-performance figures in lithium–metal battery literature. The text-mining engine can parse data from relevant paragraphs, categorize information, and extract values from the text, while the graph-mining tool can detect and extract the data of interest from the battery-performance figures. Text and graph data were matched to construct a material-property database of 8,074 cells with an accuracy of 0.989 and an F1 score of 0.933.

By treating both text and figures as data sources, the LLMB workflow comes closer to how scientists extract knowledge from papers in practice.

Moving beyond data extraction, the authors leveraged the database to train machine learning models that relate battery components to performance outcomes. Importantly, these models are paired with molecular-level descriptors to provide mechanistic interpretability. The model revealed that weakly solvating electrolytes, associated with low EState VSA6 values, a molecular descriptor capturing aspects of electronic structure and molecular shape, favor the formation of anion-derived solid-electrolyte interphase and more crystalline lithium plating. This insight is then tested experimentally using Li||NCM811 coin cells with three ether solvents, which are diethyl ether (DEE), dipropyl ether (DPE), and diethylene glycol dimethyl ether (DEGDME). DEE and DPE, which are less strongly solvating than DEGDME, deliver higher initial capacity and more stable cycling performance, completing a compelling loop from literature mining to prediction and validation.

The work offers a persuasive vision of how AI could reshape scientific discovery. At the same time, several limitations merit consideration. First, the literature corpus is restricted to a single publisher, raising the possibility of publisher-specific formatting bias and potentially missing influential work published elsewhere. Second, the model is inevitably shaped by publication bias. Because the literature tends to emphasize successful results over failed ones, the data set may skew optimiztic and underrepresent negative outcomes. Third, standardizing heterogeneous literature data requires simplifying assumptions. For example, the authors used the fifth cycle as a proxy for initial capacity, given inconsistencies in how activation cycles are reported in the literature. While reasonable, this choice underscores that mined data sets are not neutral reflections of reality, but curated representations shaped by practical decisions.

More fundamentally, the study highlights the persistent challenge of capturing structurally complex battery components through literature mining. In the case of lithium–sulfur batteries, for example, the authors note that key information about sulfur host materials is often reported in forms that are insufficiently structured for effective model use, pointing to a need for better representations and reporting standards. Furthermore, critical operating parameters, such as the N/P ratio (the ratio of negative to positive electrode capacity), are sparsely reported. In these cases, the limitation is not the AI system but the underlying data landscape: models cannot learn from information that is absent or ambiguously defined.

If AI is to become a reliable partner in scientific discovery and solve real-world challenges, the scientific community may need to rethink how data is reported.

If AI is to become a reliable partner in scientific discovery and solve real-world challenges, the scientific community may need to rethink how data is reported. The limitations encountered by the LLMB agent underscore a critical need for standardized scientific reporting of both positive and negative results.^5^ Reporting their data (both positive and negative), physical structures, and operating conditions in universal, machine-readable formats and transparent ways would substantially improve the reliability and utility of future AI systems (Figure 2). In this sense, tools like LLMB do more than extract knowledge; they expose the structural weaknesses of the current literature ecosystem.

**Figure 2:**
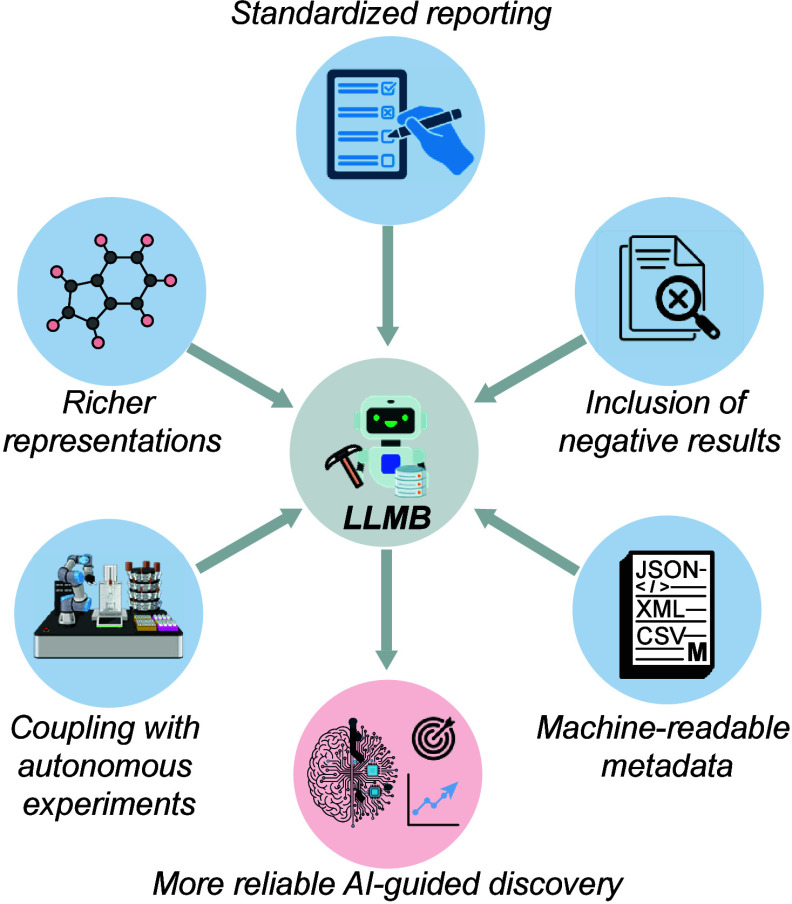
Future directions for more reliable AI-guided discovery.

Coupled with advances in automation and high-throughput experimentation, such systems suggest a future in which AI not only reads the literature more broadly than scientists can but also identifies patterns, proposes hypotheses, and helps guide experimental design.

The LLMB agent reported by Kim and co-workers is not a complete solution to battery discovery, but it represents a meaningful step toward integrating AI into the scientific workflow. Coupled with advances in automation and high-throughput experimentation, such systems suggest a future in which AI not only reads the literature more broadly than scientists can but also identifies patterns, proposes hypotheses, and helps guide experimental design. That shift, from passive repository to active partner, marks an important evolution in how knowledge is generated and used.
